# Sanger and Next-Generation Sequencing data for characterization of CTL epitopes in archived HIV-1 proviral DNA

**DOI:** 10.1371/journal.pone.0185211

**Published:** 2017-09-21

**Authors:** Camille Tumiotto, Lionel Riviere, Pantxika Bellecave, Patricia Recordon-Pinson, Alice Vilain-Parce, Gwenda-Line Guidicelli, Hervé Fleury, Provir/Latitude 45 collaborating group

**Affiliations:** 1 Laboratoire de Virologie, CHU de Bordeaux et CNRS UMR 5234, Bordeaux, France; 2 Laboratoire d’Immunologie, CHU de Bordeaux, Bordeaux, France; Universita degli Studi di Roma Tor Vergata, ITALY

## Abstract

One of the strategies for curing viral HIV-1 is a therapeutic vaccine involving the stimulation of cytotoxic CD8-positive T cells (CTL) that are Human Leucocyte Antigen (HLA)-restricted. The lack of efficiency of previous vaccination strategies may have been due to the immunogenic peptides used, which could be different from a patient’s virus epitopes and lead to a poor CTL response. To counteract this lack of specificity, conserved epitopes must be targeted. One alternative is to gather as many data as possible from a large number of patients on their HIV-1 proviral archived epitope variants, taking into account their genetic background to select the best presented CTL epitopes. In order to process big data generated by Next-Generation Sequencing (NGS) of the DNA of HIV-infected patients, we have developed a software package called TutuGenetics. This tool combines an alignment derived either from Sanger or NGS files, HLA typing, target gene and a CTL epitope list as input files. It allows automatic translation after correction of the alignment obtained between the HxB2 reference and the reads, followed by automatic calculation of the MHC IC_50_ value for each epitope variant and the HLA allele of the patient by using NetMHCpan 3.0, resulting in a csv file as output result. We validated this new tool by comparing Sanger and NGS (454, Roche) sequences obtained from the proviral DNA of patients at success of ART included in the Provir Latitude 45 study and showed a 90% correlation between the quantitative results of NGS and Sanger. This automated analysis combined with complementary samples should yield more data regarding the archived CTL epitopes according to the patients’ HLA alleles and will be useful for screening epitopes that in theory are presented efficiently to the HLA groove, thus constituting promising immunogenic peptides for a therapeutic vaccine.

## Introduction

Human Immunodeficiency Virus type 1 (HIV-1) induces a chronic infection leading to immunosuppression. While viral replication can be controlled by anti-retroviral treatment (ART), the latter cannot be interrupted since this would lead in most cases to a rapid viral rebound [1]. The next step is viral cure for which there are various strategies [2], therapeutic vaccination of patients under treatment being particularly promising. This vaccination involves the stimulation of a cytotoxic CD8-positive T cell (CTL)-mediated immune response which should be able to target the virus within the latent viral reservoir [3]. CTL epitopes are Human Leucocyte Antigen (HLA)-restricted and must be presented by molecules encoded by HLA alleles, thereby introducing a genetic and individual parameter into the physiopathology of the immune response and the evolution of HIV disease [4,5]. Some studies have shown that the viruses recovered from archived DNA are different from pre-ART circulating viruses and [6]. Therefore the characterization of CTL epitopes encoded by archived DNA in patients according to their HLA alleles should generate information on epitopes that could be used for therapeutic vaccination in future trials.

Provir Latitude 45 is a project aiming at identifying CTL epitopes in archived HIV-1 DNA in patients during ART, taking into account their HLA A and B genotype. It was initiated in Bordeaux University Hospital and then extended to other groups (Montreal, Canada and Lima, Peru). It aims at gathering as broad a dataset as possible on archived epitope sequences from a large number of patients and at identifying epitopes that are conserved and potentially used for vaccination, taking into account the patients’ genetic background.

The present study concerned the sequence analysis of archived HIV-1 DNA using Sanger technology and Next-Generation Sequencing (NGS). As previously detailed [7], the lack of frame awareness in the alignments in the Amplicon Variant Analyzer (AVA) software provided by Roche and the frequent shifts due to homopolymers complicates the analysis of amino acid sequences generated by 454 Roche sequencing. Therefore, we have developed software able to perform an automatic analysis of nucleic acid sequences of Gag and Pol among other genes, leading to the identification of CTL epitopes and MHC IC_50_ values calculated from the Immune Epitope DataBase (IEDB) according to patients’ specific HLA A and B alleles. This software can be used for the population analysis of archived epitopes using either Sanger or NGS technology. It was validated in this study by comparing both sequencing strategies with Sanger as the reference.

## Methods

### Study patients

Thirty-five patients were recruited at Bordeaux University Hospital, France, 6 patients from the primary infection cohort in Montreal, Canada and 6 patients at IMPACTA, Lima, Peru. All had been treated successfully with antiretroviral drugs for at least 6 months and all had viral loads below the threshold of available local molecular assays (< 40 copies then < 20 copies/mL in Bordeaux, < 40 copies/mL in Montreal and < 400 copies/mL in Lima). The study received authorization from the “Comité de protection des personnes du Sud-Ouest” (DC 2012/48).

### DNA extraction, Gag and Pol PCR amplification and Sanger sequencing

Total whole blood was collected from the patients and DNA was extracted from the PBMC followed by amplification and Sanger sequencing as described previously [4,5]. Sanger sequences are available in GenBank under accession numbers MF580925 to MF580945.

### Gag and Pol Ultra Deep Pyro Sequencing (UDPS)

Gag and Pol ultra-deep-sequencing were performed by using the Roche 454 technology. Amplicons for Gag (obtained with the same primers as for Sanger sequencing) and Pol [4,5] were pooled at equimolar concentrations. Clonal amplification on beads (EmPCR) was performed by using the 454 Life Science Reagents that enable bi-directional sequencing, composed of 30 cycles of PCR amplification. DNA-containing beads were recovered and UDPS was performed on the GS Junior Sequencer (454 Life Science Roche). Analysis of the obtained data is detailed below. Raw data are available in GenBank under accession numbers SRP114367.

### HLA class I typing

The method used for the molecular characterization of HLA alleles A and B in the Bordeaux and Montreal cohorts has been described previously [4,5]. For the patients from Lima, high-resolution HLA typing was performed by Sequence-Based Typing (SBT) at the University of Oklahoma Health Science Center, a CLIA/ASHI-accredited HLA typing laboratory, using in-house methods. Briefly, genomic DNA was extracted from PBMCs using a QIAamp DNA blood kit (QIAGEN). After confirmation, the PCR product was purified using an ExoSAP-IT kit (USB) and was sequenced using BigDye® Terminator v3.1 (APPLIED BIOSYSTEMS) chemistry. Dye was removed by ethanol precipitation. Sequencing reactions were performed on a 3730 Capillary Electrophoresis DNA Sequencer (APPLIED BIOSYSTEMS). Four-digit HLA types were determined using the HLA typing program Assign SBT (Conexio Genomics).

### Immune recognition tools

Viral epitopes were selected from the Los Alamos HIV Immunology Database using the most recent update of the optimal CTL epitope list [8]. Peptide binding to its restricting HLA class I molecule was predicted by using the NetMHCpan 3.0 software from the Immune Epitope Database (IEDB) [9]. A value of 500 nM for the MHC IC50 (IC50) was used as the threshold between binder and non-binder epitopes and their variants according to the recommendation given by IEDB and other groups [10–14].

### NGS sequence data analysis

Analysis of reads was performed using a part of the AVA Software pipeline. After de-multiplexing, reads obtained for a sample were grouped and aligned with the HxB2 reference [7]. The alignment of the individual reads was then recovered from AVA. UDPS is known to generate errors of around 1% [15,16]. These errors are mostly insertions and deletions and occur especially in homopolymeric areas. Consequently, read alignments generate gaps and cannot be translated directly. As stated in [7], analysis of raw UDPS data is not possible using only the AVA pipeline. The nucleotide alignment cannot be translated directly without correction because of the lack of frame awareness of AVA. Since our goal was to analyze the CTL epitopes archived in the proviral HIV-1 DNA, we needed to translate the reads into amino-acid sequences and then calculate the MHC IC50 score for each epitope/HLA combination. This involves:

(i) having as accurate a translation as possible to produce an in-frame correct amino acid sequence; (ii) retrieving the CTL epitopes described in the Best-Defined CTL Epitopes List (BDCEL) [8] (https://www.hiv.lanl.gov/content/immunology/tables/optimal_ctl_summary.html); (iii) calculating the MHC IC50 of the epitopes using the MHC-I Processing Prediction from IEDB (http://tools.immuneepitope.org/processing/), considering all combinations of HLA alleles, peptide size, epitope and variant sequences; and then (iv) merging the results from each step into a single spread-sheet for further analysis. Performing all these steps manually is time-consuming and subject to error.

### TutuGenetics software

**Fig 1:**
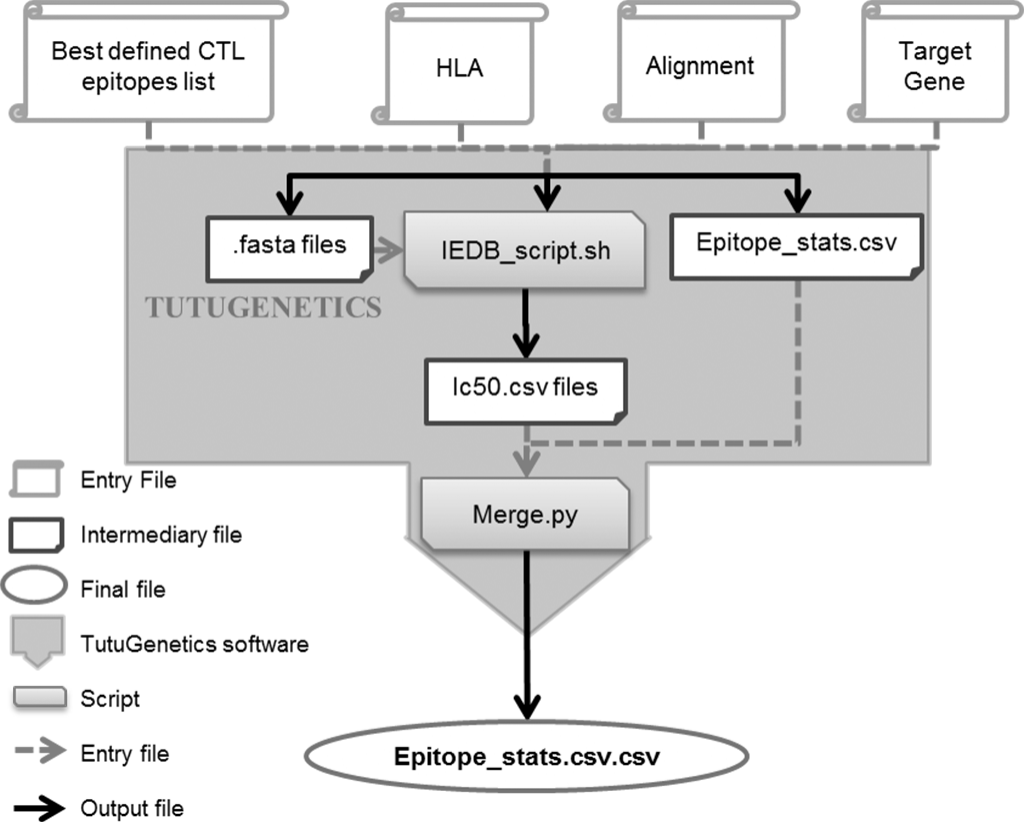
Description of TutuGenetics software. Four input files are used to generate an output final file merging the quantification (% variants) and qualification (amino-acid sequence) of the variability of HIV epitopes as well as their theoretical presentation to their HLA molecules (IC_50_ value in nM). It allows the automated analysis of nucleic acid alignments from Sanger or UDPS sequences, corrects them (for the UDPS) and translates each read into its corresponding amino acids. Then, according to the HLA genotype of the patient, it retrieves the CTL epitopes and their variants according to the Los Alamos HIV Immunology Database CTL epitope list. Finally, given the patient’s HLA, the IC_50_ scores of each epitope and their variants are calculated using the stand-alone of the NetMHCpan 3.0 software.

TutuGenetics is a command line software tool written in C taking as inputs numerous files “Fig 1”:

- The BDCEL which list HIV-1 epitopes described for given HLA alleles.
- The patient’s HLA alleles A and B.
- HxB2 and the patients’ sequences alignment.
- The specified target gene required to determine the correct open reading frame for the amino-acid translation.

As stated earlier, the UDPS alignment cannot be translated directly. Prior to translation, TutuGenetics is able to rearrange the UDPS alignment according to some filtering criterion such as a minimum reads length and alignment correction heuristic.

The alignment issued from a Sanger sequencing or the corrected UDPS are then translated into amino acid sequences given the open reading frame (ORF) of the gene specified by the user.

Based on translated reads/sequences, a given gene and a given HLA allele, TutuGenetics collects epitopes and their variants defined for the corresponding HLA it results in a file that qualifies and quantifies the genetic variability of the selected epitopes. It also generates a shell script instrumenting the stand-alone NetMHCpan 3.0 program from the IEDB for the MHC-I Processing Prediction. This NetMHCpan 3.0 program calculates the MHC IC_50_ score for each epitope according to the patient HLA allele. Then, TutuGenetics merges all the results in a single spread-sheet.

The output file is a csv file summarizing the HLA alleles, the position of the epitope relative to the HxB2 reference, the epitope sequence, the number of reads encoding this epitope, the percentage of reads containing this epitope and the MHC IC_50_ value_._ The first line of an epitope described for an HLA allele is the reference HxB2 and its corresponding IC_50_.

## Results

### Patients’ characteristics

To date, the analysis of archived proviral HIV-1 DNA in Sanger and UDPS has been obtained for 22 patients of the 47 included in this study. The comparison has been done in 19 patients for Pol and in 12 for Gag. Table 1 summarizes the characteristics of the viral clades and HLA alleles for the 22 patients.

**Table 1:** Patients’ characteristics. The viral clade and HLA allele A and B typing results for each individual from the French, Canadian or Peruvian cohort are presented.

Sample ID	Geographic origin	Viral Clade	HLA allele
B01_Bg	Bordeaux	B	A*02:01 B*15:01 B*44:02
B02_Bl	Bordeaux	B	A*02:01 A*29:02 B*27:05 B*44:03
B03_Ba	Bordeaux	B	A*02:01 A*24:02 B*27:05 B*51:01
B04_Ce	Bordeaux	B	A*02:01 A*29:02 B*39:01 B*44:03
B05_Ca	Bordeaux	B	A*02:01 A*02:22 B*14:01 B*15:01
B06_Cc	Bordeaux	B	A*02:01 A*26:01 B*08:01 B*38:01
B07_Dj	Bordeaux	CRF02_AG	A*02:01 A*68:01 B*15:02 B*51:01
B08_Jc	Bordeaux	B	A*02:01 A*30:01 B*13:02 B*40:01
B09_Ma	Bordeaux	CRF01_AE	A*01:01 A*02:01 B*35:03 B*44:02
B10_Mj	Bordeaux	B	A*03:01 A*68:01 B*07:02 B*44:02
B11_Sy	Bordeaux	B	A*02:01 A*26:01 B*39:01 B*40:01
B12_Sb	Bordeaux	A1	A*02:01 A*02:05 B*15:03 B*18:01
B13_TEp	Bordeaux	B	A*02:01 B*40:01 B*44:02
B14_TIp	Bordeaux	B	A*01:01 A*02:01 B*07:02 B*51:01
B15_Vp	Bordeaux	B	A*01:01 A*02:01 B*44:02 B*57:01
M01_A4	Montreal	B	A*02:01 A*33:03 B*50:01 B*58:01
M02_A7	Montreal	B	A*03:01 A*30:02 B*07:02 B*18:01
M03_G0	Montreal	B	A*02:01 A*24:02 B*08:01 B*18:01
M04_G1	Montreal	B	A*02:01 A*29:02 B*08:01 B*44:03
M05_H9	Montreal	B	A*01:01 B*35:01 B*51:01
L01_H6	Lima	B	A*24:02 A*29:02 B*35:10 B*44:03
L02_H0	Lima	B	A*24:02 B*35:43 B*58:01

Comparison between theoretical presentations of CTL epitopes archived in proviral DNA for the sequences obtained with Sanger and NGS:

To validate our tool TutuGenetics, we focused on epitopes described on the BDCEL. As Sanger sequencing is the gold standard method for describing HIV sequences, NGS results were compared to Sanger sequencing results after TutuGenetics processing.

TutuGenetics provided the following: sample name, HLA allele, position of examined peptide in Gag, RT or Protease proteins, amino-acid sequence of epitope referenced in HxB2 (first lane) and potential variants (the following lanes), theoretical MHC IC_50_ value (nM) according to IEDB, number of reads encoding the epitope and fulfilling TutuGenetics criteria as described above and their percentages. The last column indicates the presence or absence of the different epitopes in the sequences obtained by the Sanger method. The threshold of 1% for the detection of variants was determined according to previous data from our group [17].

Globally we analyzed 133 Gag or Pol epitopes. The results obtained with Sanger sequencing and AVA combined with TutuGenetics data were concordant for 120/133 epitopes (90.2%). Results obtained for 8 samples are presented in Table 2.

**Table 2:** Output of TutuGenetics and comparison with Sanger sequencing results: Eight HLA/peptide associations are detailed. For each sample and HLA, the first line shows the putative epitope described in the HxB2 reference sequence and the calculated IC50 (nM). For each epitope, the number of NGS reads as well as the percentage of variants is also shown. The mutated amino-acids are in bold. In the last column, “yes” means that this epitope obtained with NGS was retrieved in the Sanger sequence; “no” indicates that the epitope can be quantified by NGS but was not observed by Sanger.

Sample ID	HLA	Subprotein	Epitope	Ic50 (nM)	nb reads	% variants	Concordance with Sanger
***HxB2***	HLA-B*27:*05*	***p17 (19–27)***	***IRLRPGGKK***	***192*.*5***			
B03_Ba	HLA-B*27:05	p17 (19–27)	IRLRPGGKK	192.5	4949	93%	yes
B03_Ba	HLA-B*27:05	p17 (19–27)	**L**RLRPGGKK	144.5	71	1.3%	no
***HxB2***	HLA-B*15:*01*	***p24 (137–145)***	***GLNKIVRMY***	***392*.*9***			
B01_Bg	HLA-B*15:01	p24 (137–145)	GLNKIVRMY	392.9	8336	96.8%	yes
B01_Bg	HLA-B*15:01	p24 (137–145)	**E**LNKIVRMY	1467.1	108	1.3%	no
***HxB2***	HLA-A*24:*02*	***p17 (28–36)***	***KYKLKHIVW***	***256*.*9***			
M03_G0	HLA-A*24:02	p17 (28–36)	**Q**YKLKH**L**VW	405	896	89.8%	yes
M03_G0	HLA-A*24:02	p17 (28–36)	**Q**YKLKHIVW	517.9	53	5.3%	no
***HxB2***	HLA-B*40:*01*	***p24 (44–52)***	***SEGATPQDL***	***492*.*6***			
B13_TEp	HLA-B*40:01	p24 (44–52)	SEGATPQDL	492.6	1160	76.5%	yes
B13_TEp	HLA-B*40:01	p24 (44–52)	SE**R**ATPQDL	600.9	312	20.6%	no
***HxB2***	HLA-B*40:*01*	***p2p7p1p6 (118–126)***	***KELYPLTSL***	***16*.*8***			
B13_TEp	HLA-B*40:01	p2p7p1p6 (118–126)	KELYPL**A**SL	14.7	2603	94.9%	yes
B13_TEp	HLA-B*40:01	p2p7p1p6 (118–126)	KELYPL**AP**L	11.9	38	1.4%	no
B13_TEp	HLA-B*40:01	p2p7p1p6 (118–126)	**E**ELYPL**A**SL	51.7	31	1.1%	no
***HxB2***	HLA-A*29:*02*	***p17 (78–86)***	***LYNTVATLY***	***25***			
B04_Ce	HLA-A*29:02	p17 (78–86)	L**F**NTVATLY	12.1	5315	94.4%	yes
***HxB2***	HLA-A*24:*02*	***p17 (28–36)***	***KYKLKHIVW***	***256*.*9***			
L01_H6	HLA-A*24:02	p17 (28–36)	**Q**Y**R**LKHIVW	528.9	1360	88.3%	yes
L01_H6	HLA-A*24:02	p17 (28–36)	**E**Y**R**LKHIVW	1437.3	26	1.7%	no
L01_H6	HLA-A*24:02	p17 (28–36)	**Q**Y**S**LKHIVW	244.2	49	3.2%	no
L01_H6	HLA-A*24:02	p17 (28–36)	**E**Y**S**LKHIVW	850.8	16	1%	no
***HxB2***	HLA-B*40:*01*	***p17 (11–19)***	***GELDRWEKI***	***358*.*3***			
B11_Sy	HLA-B*40:01	p17 (11–19)	G**K**LD**K**WE**R**I	20805.1	2114	95.4%	yes
***HxB2***	HLA-B*35:*01*	***RT (118–127)***	***VPLDEDFRKY***	***424*.*1***			
M05_H9	HLA-B*35:01	RT (118–127)	VPLDEDFRKY	424.1	532	76.9%	yes
M05_H9	HLA-B*35:01	RT (118–127)	VPLDEDFRK**C**	29962.8	140	20.2%	no

We then analyzed for each sample the variability of the epitope sequences and their predicted binding to the patient HLA allele.

B03_Ba expresses the HLA-B*27:05 allele which allows presentation to CTL of the Gag p17 (19–27) epitope; the reference antigen sequence according to the Los Alamos database is noted in the first line. The same sequence was found in the patient’s provirus and observed by Sanger while the 454 NGS yields a percentage of 92.99% of the peptide. Interestingly, an epitope variant with a change from I to L at the first aa position was not observed by Sanger but was quantified at 1.33% by NGS. This change did not affect the predicted epitope binding to HLA-B*27:05.

B01_Bg expresses the HLA-B*15:01 allele which has been shown to present the Gag p24 (137–145) epitope, as indicated in the first line (IC_50_ = 395.9 nM). This epitope was observed by Sanger and quantified at 96.83% by NGS. Remarkably, an epitope variant with G to E mutation was detected only by NGS (1.26%) and does not theoretically bind to HLA-B*15:01.

M03_G0 expresses the HLA-A*24:02 allele associated with recognition of the Gag p17 (28–36) epitope. The sequence analysis yielded two epitopes which are different from the HxB2 peptide. One epitope was identified by both technologies and should be presented to CD8 T cells and a second one was quantified only at 5.31% by NGS and is at the limit of theoretical binding affinity for effective CTL recognition.

B13_TEp expresses the HLA B*40:01 allele with potential presentation of the Gag p24 (44–52) epitope; the reference peptide was identified by Sanger and quantified by NGS at 76.47%. An epitope variant with a predicted MHC IC_50_ above the threshold for theoretical recognition was not observed by Sanger but was quantified at 20.57% by NGS. With this same HLA allele, potential recognition for Gag p2p7p1p6 (118–126) was possible. In this case, three different epitope sequences were identified and quantified by NGS at 94.86, 1.39 and 1.13%. The sequence was also identified by Sanger and all three variants are predicted to bind HLA-B*40:01.

With HLA allele A*29:02, patient B04_Ce could present the Gag p17 (78–86) epitope. Although exhibiting a Y to F mutation compared to the Hxb2 reference, the peptide encoded by provirus should still be presented. The sequence was detected by Sanger while quantified at 94.42% by NGS.

Patient L01_H6 expresses the HLA A*24:02 allele which can present the Gag p17 (28–36) epitope. The reference peptide was not observed in the archived virus but four mutated variants were quantified by NGS. Only the variant detected at 88.25% was found by Sanger. Three of the four variants had MHC IC_50_ values above the threshold for recognition, while the fourth was detected at a very low level and is predicted to bind HLA-A*24:02.

B11_Sy expresses HLAB*40:01 with theoretical presentation of Gag p17 (11–19). Compared to the HxB2 reference sequence, the epitope was present in archived provirus containing two aa mutations found both by Sanger and NGS (95.4%). These changes affect binding to the HLA molecule since its MHC IC_50_ corresponds to 20805 nM (compared to IC_50_ 358.3 nM for HxB2).

Patient M05_H9 expresses HLA-B*35:01 and should be able to present the RT (118–127) epitope. An epitope sequence similar to our reference was noted by Sanger with a NGS quantitation at 76.88%. An epitope variant which is theoretically unable to bind to HLA-B*35:01 was not detected by Sanger but was quantitated by NGS (20.23%)

## Discussion

TutuGenetics allows the rapid and reliable analysis of NGS data with direct and HLA allele-dependent identification of the CTL epitope according to a reference HIV-1 epitope list and predication of epitope binding affinity. The correlation between the quantitative results of NGS and Sanger was high and in accordance with a threshold of variant detection around 20 to 30% for NGS quantitation/Sanger detection, as generally admitted in the field [18]. This tool is thus able to facilitate the identification of archived CTL epitopes according to the patients’ HLA alleles for their potential inclusion in therapeutic vaccine immunogens.

As already pointed out by Deng et al [6], a large number of archived HIV-1 DNA genomes carry CTL epitope escape mutations even in patients close to primary infection. It renders infected cells insensitive to CTL directed at conserved/wild type epitopes when ART is interrupted. It is therefore crucial to identify CTL epitopes that are non-mutated or, if they do show sequence mutations, that have retained their ability to bind to the presenting HLA class I molecule. The pattern is very complicated since data indicate that a process of viral adaptation to high-frequency HLA alleles can occur in a given population [19], therefore conferring an advantage to some rare HLA supertypes in HIV disease progression [20].

TutuGenetics may be able to overcome this issue by generating big data from a large number of patients with the hypothesis that some archived CTL epitopes cannot vary for viral functional reasons, even though they are under pressure from the most frequent HLA alleles in a population. One example of a study similar to Provir but devoted to the analysis of a preventive vaccine trial (RV144) has shown that there is an association between vaccine efficacy and HLA A*02:01 [21]. TutuGenetics has the advantage to be modular. Therefore it accepts as input data files generated by other NGS platforms (PGM Ion Torrent and Illumina). Also, the search of epitopes may not be restricted to the BDCEL list and can be used to identify novel epitopes for other viruses.

Some important issues remain to be solved. One is the variability of these epitopes within the quasi-species. This variability, if present, is mainly measurable by NGS; if there is a high variability of the CTL epitopes within the quasi-species with some variants outside the range of theoretical presentation, it will be more complicated to design a peptide cocktail reacting with the most frequent HLA alleles. As noted above, most of the DNA sequences that are studied by NGS carry defective proviruses and do not allow identification of the viruses that will resume replication at ART interruption [22]. The only way to study a virus resuming from the archived DNA is to induce replication in cell culture using memory TCD4 cell-enriched samples. However, these techniques require large volumes of blood and clearly do not reflect the full range of the viral reservoir [6]. Moreover, they do not allow the investigation of the numerous patients required by such a strategy, in which the quest would be to define highly frequent conserved archived epitopes able to be presented by the most common HLA alleles given the above mentioned theoretical limitation. Sequencing analysis also has its limitations. First, sequenced full-length genomes are not available and even if they were, this would not necessarily mean that they indicate that the genomes come from single viruses: they may arise from computerized alignments of parts of different genomes covering the full length of the virus. To overcome these limitations, an even more labor-intensive single-genome sequencing approach would need to be employed. However, since T cells are reactive with short, linear epitopes, the issue of wrongful reconstruction of full-length genomes may not have a dramatic effect.

The second issue is the variability of the CTL epitopes observed in different compartments, particularly PBMC and gut-associated lymphoid tissues (GALT) [23]. While some studies seem to indicate that the viral reservoir in patients on long-term suppressive ART is stable with few genetic changes [24], it would still be necessary to check by NGS whether such a conclusion would be applicable to CTL epitopes of archived virus in various compartments. If the corresponding CTL epitopes were to be different from one compartment to the other, this would add an additional hurdle to cross. Further studies are thus needed to investigate these issues. They will be facilitated by our TutuGenetics software, which can use raw data from different NGS sequencers, namely the PGM Ion Torrent and the Illumina.
